# Tumor Immune Microenvironment and Genetic Alterations in Mesothelioma

**DOI:** 10.3389/fonc.2021.660039

**Published:** 2021-06-23

**Authors:** Stefanie Hiltbrunner, Laura Mannarino, Michaela B. Kirschner, Isabelle Opitz, Angelica Rigutto, Alexander Laure, Michela Lia, Paolo Nozza, Antonio Maconi, Sergio Marchini, Maurizio D’Incalci, Alessandra Curioni-Fontecedro, Federica Grosso

**Affiliations:** ^1^ Department of Medical Oncology and Hematology, University Hospital Zurich, Zurich, Switzerland; ^2^ Comprehensive Cancer Center Zurich, University of Zurich, Zurich, Switzerland; ^3^ Department of Oncology, Istituto di Ricerche Farmacologiche Mario Negri Istituto di Ricovero e Cura a Carattere Scientifico (IRCCS), Milano, Italy; ^4^ Department of Thoracic Surgery, University Hospital Zurich, Zurich, Switzerland; ^5^ Mesothelioma Unit, Azienda Ospedaliera SS. Antonio e Biagio e Cesare Arrigo, Alessandria, Italy; ^6^ Department of Pathology, Azienda Ospedaliera SS. Antonio e Biagio e Cesare Arrigo, Alessandria, Italy; ^7^ Infrastruttura Ricerca Formazione Innovazione (IRFI), Dipartimento Attività Integrate Ricerca e Innovazione (DAIRI), Azienda Ospedaliera SS. Antonio e Biagio e Cesare Arrigo, Alessandria, Italy; ^8^ Translational Medicine, Dipartimento Attività Integrate Ricerca e Innovazione (DAIRI), Azienda Ospedaliera SS. Antonio e Biagio e Cesare Arrigo, Alessandria, Italy

**Keywords:** mesothelioma, tumor microenvironment, genetic alterations, immunotherapy, targeted therapy

## Abstract

Malignant pleural mesothelioma (MPM) is a rare and fatal disease of the pleural lining. Up to 80% of the MPM cases are linked to asbestos exposure. Even though its use has been banned in the industrialized countries, the cases continue to increase. MPM is a lethal cancer, with very little survival improvements in the last years, mirroring very limited therapeutic advances. Platinum-based chemotherapy in combination with pemetrexed and surgery are the standard of care, but prognosis is still unacceptably poor with median overall survival of approximately 12 months. The genomic landscape of MPM has been widely characterized showing a low mutational burden and the impairment of tumor suppressor genes. Among them, *BAP1* and *BLM* are present as a germline inactivation in a small subset of patients and increases predisposition to tumorigenesis. Other studies have demonstrated a high frequency of mutations in DNA repair genes. Many therapy approaches targeting these alterations have emerged and are under evaluation in the clinic. High-throughput technologies have allowed the detection of more complex molecular events, like chromotripsis and revealed different transcriptional programs for each histological subtype. Transcriptional analysis has also paved the way to the study of tumor-infiltrating cells, thus shedding lights on the crosstalk between tumor cells and the microenvironment. The tumor microenvironment of MPM is indeed crucial for the pathogenesis and outcome of this disease; it is characterized by an inflammatory response to asbestos exposure, involving a variety of chemokines and suppressive immune cells such as M2-like macrophages and regulatory T cells. Another important feature of MPM is the dysregulation of microRNA expression, being frequently linked to cancer development and drug resistance. This review will give a detailed overview of all the above mentioned features of MPM in order to improve the understanding of this disease and the development of new therapeutic strategies.

## Introduction

Malignant pleural mesothelioma (MPM) is an aggressive malignancy of the pleural lining with limited treatment options. It is strongly associated with exposure to fibrous material such as asbestos. Due to the long latency period of up to 40 years and the ongoing use of asbestos in developing countries, the cases are still rising. Patients with MPM have a very short median overall survival of around 12 months after diagnosis and are treated with a combination of surgery, radiotherapy and chemotherapy. Pharmacological treatment has not changed for years consisting in the combination of cisplatin with pemetrexed and/or bevacizumab in some cases. Studies performed so far deciphered the genomic, transcriptional and epigenomic landscape of MPM, highlighting a complex and not yet known scenario. Very recently, the combination of two immune checkpoint inhibitors showed an improvement in overall survival compared to standard chemotherapy in first line. Nevertheless, current therapies have not improved, there is no second line therapy available and inclusion into clinical trials is currently the best option. The tumor microenvironment (TME) of mesothelioma consists of a wide variety of innate and adaptive immune cell subtypes, stromal and endothelial cells and has been characterized as a highly inflammatory TME favoring treatment with immune checkpoint inhibitors. On the other hand, mesothelioma is considered a non-immunogenic cancer due to a low tumor mutational burden and paucity of activated T cells. Thus, the understanding of the crosstalk and interactions of immune, stromal and tumor cells is of major importance for the development of novel therapies and the discovery of new therapeutic targets.

## Inflammatory Tumor Microenvironment

**Figure 1:**
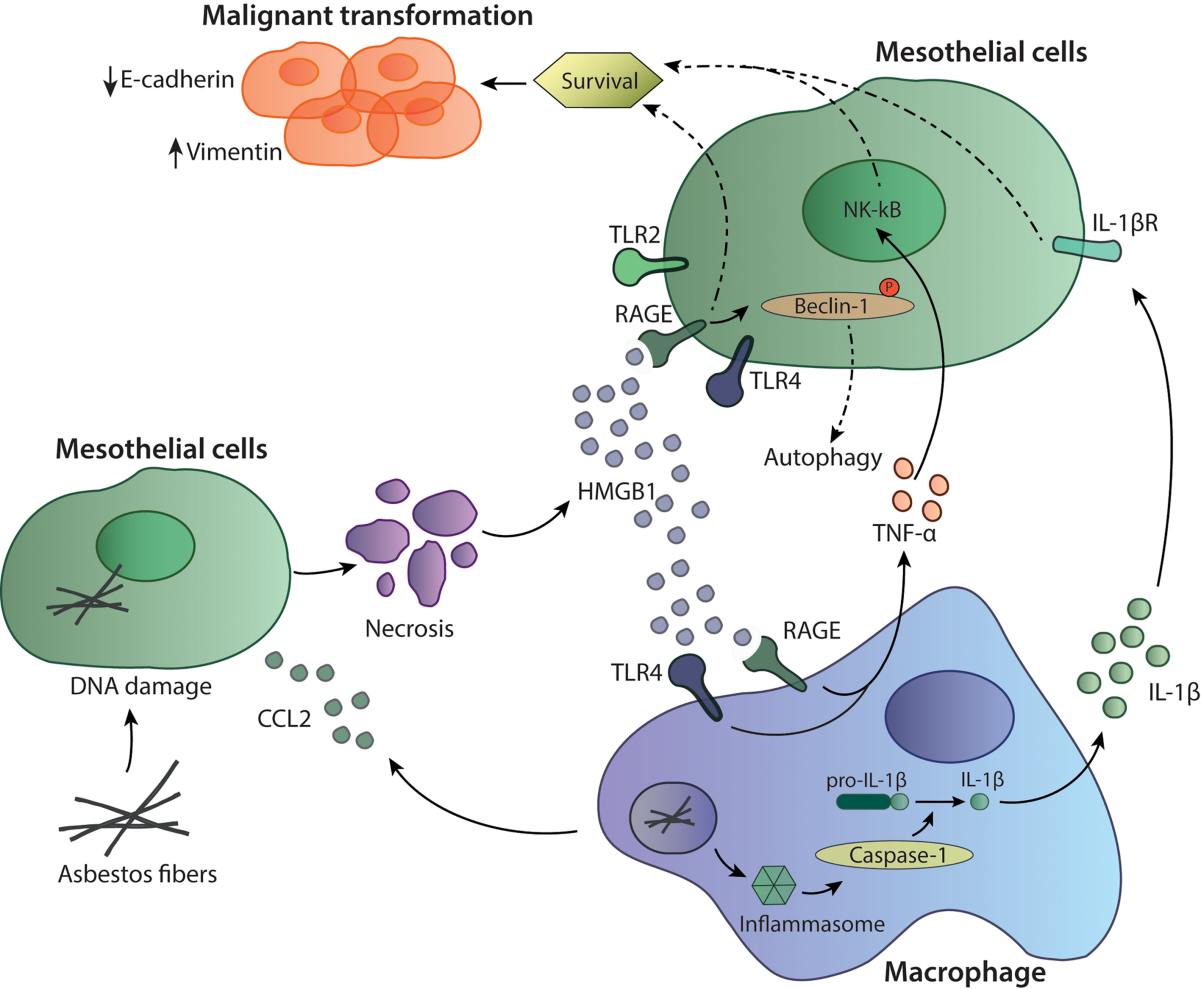
Mechanisms of asbestos-induced carcinogenesis. Asbestos fibers reach the mesothelial cells where they can induce cell death and the release of inflammatory mediators such as HMGB1 and CCL2. The recruited macrophages are activated through HMGB1 binding to TLR4 and RAGE to induce TNF-α or by inflammasome activation through asbestos fibers. Activation of caspase-1 and cleavage of pro-IL-1β to the active form IL-1β can lead to further survival signals in mesothelial cells. HMGB1 can also bind to TLR4 and RAGE expressed on mesothelial cells supporting survival of those cells.

Inhaled mineral fibers traveling to the visceral pleura and deposition of mineral fibers in the pleural lining leads to a permanent innate stimuli with subsequent chronic inflammation, production of oxygen radicals and necrotic cell death of mesothelial cells. Asbestos fibers are biopersistent and non-degradable, which plays an important role in their carcinogenic potential (1). Mesothelial cells exposed to asbestos fibers secrete C-C chemokine ligand 2 (CCL2), which attracts macrophages to the site (2). Reactive-oxygen species induce DNA damage and mutations in mesothelial cells (3) leading to necrotic cell death and to the production and release of damage-associated molecular patterns (DAMPs) including High Mobility Group Box 1 protein (HMGB1). HMGB1 is translocated from the nucleus to the cytoplasm and secreted into the extracellular space, where it can bind to its receptors TLR2, TLR4 and receptor for Advanced Glycation Endproducts (RAGE). The binding of HMGB1 to mesothelial cells enhances their proliferation and migration capacity. The release of HMGB1 also promotes autophagy, allowing a higher fraction of mesothelial cells to survive asbestos exposure. HMGB1 silencing was shown to inhibit autophagy and to increase asbestos-induced mesothelial cell death, thereby decreasing asbestos induced transformation (4) (**Figure 1**). The importance of HMGB1 in cancerous transformation was also studied in a mouse mesothelioma model, where the investigators demonstrated that inhibition of HMGB1 binding to its receptors led to decreased tumor growth (5, 6) again pointing out the importance of this mediator in MPM progression. In addition, serum concentrations of HMGB1 are also significantly higher in mesothelioma patients compared to healthy controls, indicating its significance in tumor development (7).

Recruited macrophages phagocyte asbestos fibers leading to secretion of proinflammatory mediators such as TNF-α, supporting carcinogenesis and cancer cells survival (8). On the other hand, asbestos itself can also activate the inflammasome, a multiprotein complex part of the innate immune system, leading to activation of caspase-1 and cleavage of pro-IL-1β to IL-1β (9). IL-1β released by tumor-associated macrophages (TAMs) and its binding to IL-1R on mesothelial cells can be part of the malignant phenotype inducing cell survival and proliferation (10). Furthermore, production of TNF-α and IL-1β by macrophages can also be induced through the inflammatory environment and the presence of extracellular HMGB1, which protects mesothelial cells from asbestos-induced cell death (11, 12). TNF-α released by macrophages signals through NF-κB in mesothelial cells and supports their survival to asbestos exposure (13). Thus, TNF-α and IL-1β are important players in the transformation of non-tumorigenic mesothelial cells (14). Interestingly, HMGB1 was also shown to play an important role in epithelial to mesenchymal transition (EMT) as it led to upregulation of the EMT markers vimentin and α-smooth muscle actin (12). HMGB1 can also reduce expression of E-cadherins, an epithelial marker and upregulates mesenchymal markers promoting EMT (12). Altogether, this indicates the importance of HMGB1, TNF-α and IL-1β in mediating mesothelioma malignant transformation and progression (**Figure 1**).

The mesothelioma tumor microenvironment consists of a complex structure of stromal cells, immune cells and vasculature. All of these components result in a heterogeneous plethora of possible mesothelioma phenotypes, making this disease very difficult to treat. The immune compartment is characterized by the presence of many regulatory and inhibitory cells such as regulatory T cells, type 2 macrophages and myeloid-derived suppressor cells (MDSC). Immune infiltrates also include T and B cells, NK cells, dendritic cells (DC) and neutrophils.

### NK Cells

Innate lymphoid cells (ILC) such as natural killer (NK) cells can be found in mesothelioma tumors, however, in a very small proportion (15, 16). NK cells are innate immune cells and belong to the innate lymphoid cell family (17), with high cytotoxic capacity and without the need for antigen-specific stimulation (18). NK cells are often characterized to have impaired effector functions in different solid tumors due to local immunosuppressive microenvironment leading to hampering of effector functions (19). However, little is known about the role of NK cells in mesothelioma or their possible engagement for mesothelioma therapy.

MPM tumors are infiltrated with NK cells shown by mRNA expression analysis of specific NK cell makers, which were even higher expressed in MPM compared to other cancers. However, the presence of the NK cell markers was not linked to better overall survival (20).

Similar results were obtained with staining for CD56 by immunohistochemistry (IHC) of a tissue-microarray in both epithelioid and non-epithelioid subgroups (16). In addition, different studies report different results about the expression of inhibitory or activating molecules on NK cells. A study by Nishimura et al. described that NK cells isolated from the blood of mesothelioma patients had lower cytotoxic activity compared to NK cells from healthy individuals and showed a reduced expression of the activating receptor NKp46 but normal levels of another activating receptor NKG2D (21). Another study described a higher frequency of CD56^bright^ and a lower frequency of CD56^dim^ NK cells in mesothelioma patients compared to healthy controls. Interestingly, treatment with anti-CTLA-4 immune checkpoint inhibitor changed this ratio from a higher frequency of CD56^dim^ to a more physiological level of healthy controls (20).

**Figure 2:**
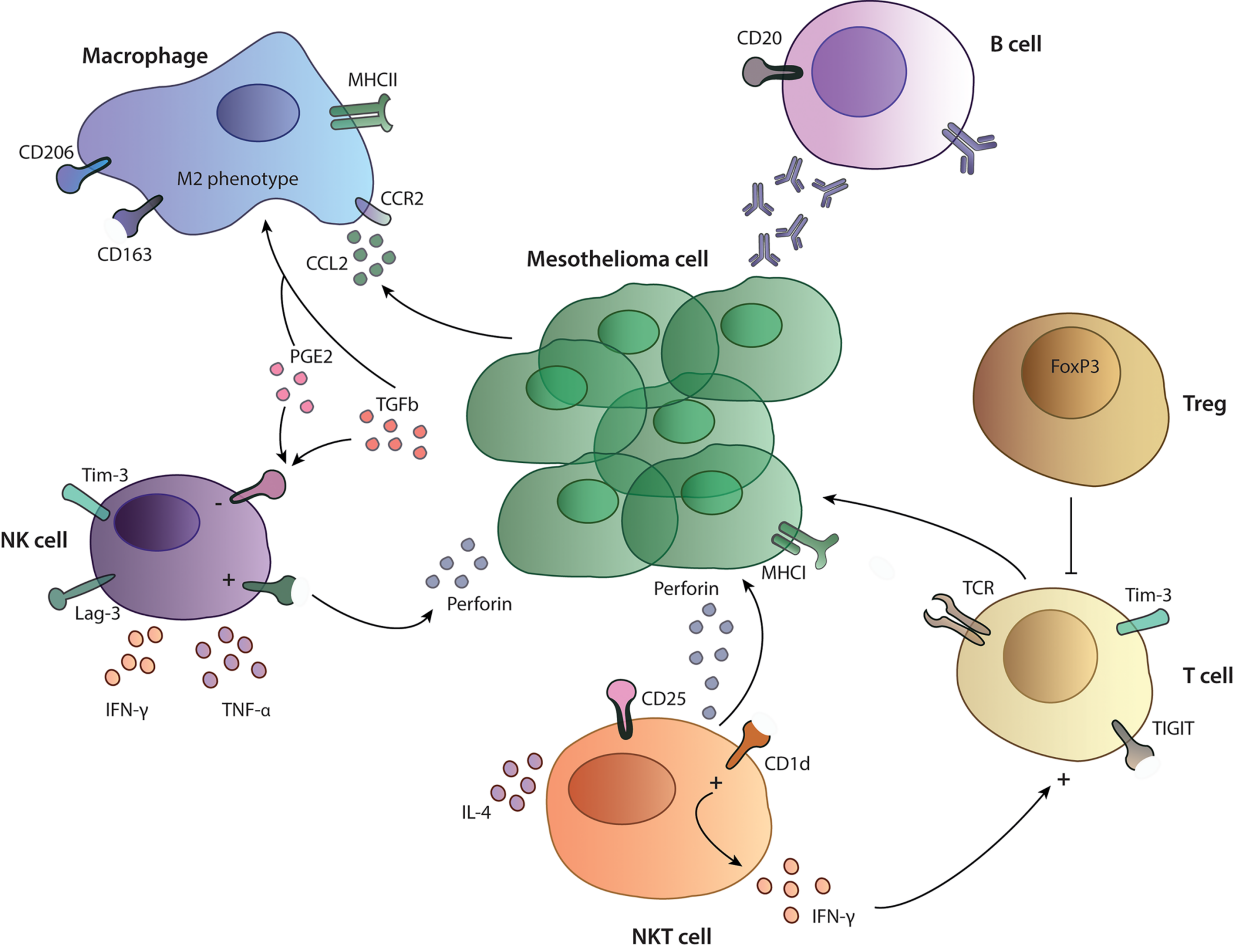
Tumor microenvironment in mesothelioma. Overview on the functionality and interactions of different immune cells studied in MPM patients. NK cells and T cells express inhibitory receptors such as TIM-3, LAG-3 and TIGIT and are influenced by a suppressive cytokines (PGE2, TGF-β) and the presence of Treg cells in performing their cytotoxic functions. Macrophages show a M2-like phenotype with expression of CD206 and CD163 on their surface. B cells in the TME produce specific antibodies against cancer cells, participating in the anti-tumor immune response.

Pleural effusion of MPM patients is often used to study the presence and function of different immune cells. NK cells isolated from pleural effusion show high expression of the immune checkpoint molecules T cell immunoglobulin and mucin-domain containing-3 (TIM-3) and lymphocyte activation gene-3 (LAG-3), whereas both molecules are expressed to the highest levels on NK cells and to a lesser extent on CD4 and CD8 T cells (22). Interestingly, LAG-3 is not expressed on MPM tumor cells (23). Here, the investigators claim that effusions are more often present in an inflammatory context, which could influence the expression of suppressive immune checkpoint molecules as they have shown that the early activation marker CD69 is significantly correlated with the expression of TIM-3. Another explanation could be that matching pleural effusion and tumor tissue do not reflect each other’s immune cell composition (24). Nevertheless, differences in effusion and tumor samples could also be due to different analysis methods (22). Furthermore, NK cells in pleural effusion from MPM patients are functional and produce high amounts of TNF-α and INF-γ upon stimulation (25) but have also an impaired expression of perforin, which can be restored by IL-2 stimulation *in vitro*. However, incubation of NK cells with pleural effusion completely abrogated the activation status of the NK cells, indicating the presence of inhibitory cytokines in the pleural effusion (26) (**Figure 2**). Similar results were obtained in a study performed by Vacca et al., they also described NK cells from pleural effusion from different cancer patients (including mesothelioma patients) as functionally capable to produce cytokines, perforin and granzyme A and B and to perform cytotoxic functions upon *in vitro* stimulation with IL-2. Furthermore, NK cells express normal levels of activating receptor including NKp30, NKp44, NKG2D, and DNAM‐1 after stimulation. This suggests a possibility for reactivation of NK cells and no expression of an anergic phenotype as described in other studies (27). However, the functional capacities of NK cells in human tumors were not investigated and it is currently unclear if they are in a state of exhaustion or can perform effector functions normally. A mouse syngenic mesothelioma model using the AE17 cell line, reveals that depletion of NK cells with an anti-asialo GM1 antibody did not influence tumor growth (28). Current data about NK cells in MPM tumors does not correlate to overall survival, nevertheless, more data is needed to understand their functional effector capacity and their exhaustion profile intratumorally and the possibility to target them with therapeutic approaches.

### NKT Cells

Natural killer T (NKT) cells are a distinct population of T cells recognizing glycolipids presented on the non-classical class I-like molecule CD1d in contrast to normal T cells, which recognize peptide fragments presented on MHC molecules (29, 30). NKT cells have lytic activity, but their main function lies in the production and secretion of a wide variety of cytokines. Upon activation, they can produce high amounts of Th1 or Th2 cytokines, which can lead to bystander activation of NK cells, CD8 T cells and dendritic cells.

Little is known about the function of NKT cells in the tumor microenvironment of patients with MPM. Altomare et al. investigated the presence of NKT cells in the blood of MPM patients. Here, they showed that MPM patients have a higher frequency of circulating NKT cells compared to healthy volunteers, whereas there were no differences in their ability to produce IFN-γ and IL-4 (31) (**Figure 2**). NKT cells have been mainly studied in the context of mesothelioma mouse models and as a therapeutic target, since they can easily be activated by artificial glycolipids. In the pleural effusion of a MPM mouse model, NKT cells are present, express high levels of the activation marker CD25 and produce large amounts of INF-γ. In the same model, activation of NKT cells through administration of glycolipids led to prolonged survival in the treated groups (32, 33), indicating that these cells have an anti-tumor phenotype and can activate other cytotoxic cells. More studies are needed in order to understand the possibility to use them as a therapeutic target in MPM.

### Macrophages

Macrophages are innate immune cells specialized in phagocytosis, engulfing and digestion of invading organisms and cell debris and play an important role in tissue homeostasis. Monocytes are recruited from the blood to the TME through locally produced chemokines and become TAMs and, in patients’ blood, increased amounts of circulating monocytes and a low lymphocyte to monocyte ratio have been reported to negatively correlate with overall survival (34, 35). TAMs can be divided into two subset depending on their function and marker expression. M1 macrophages are proinflammatory macrophages and have strong capacity to kill invading pathogens and contribute as well to tissue destruction. M2 macrophages are important mediators in tissue remodeling, allergic diseases and angiogenesis. Nevertheless, macrophages are a functionally diverse and plastic group and can reverse their polarization from M2 to M1 depending on the chemokine environment (36–38).

Various cytokines can induce differentiation of monocytes and macrophages into TAMs, such as CCL2, C–C chemokine ligand 4 (CCL4), C–C chemokine ligand 5 (CCL5) and C-X-C motif chemokine ligand 12 (CXCL12) secreted by MPM cells (39, 40). CCL2, the most studied TAM-associated chemokine, recruits immune cells such as T cells, macrophages and dendritic cells through binding to CCR2 on the cells to the inflammatory site (41). CCL2 is upregulated in pleural effusion and serum from MPM patients compared to benign pleural effusion or pleural effusion from other malignancies and serum from healthy volunteers, respectively (42–44). Interestingly, CCL2 levels also correlate with the tumor stage, indicating an important role of macrophages in disease progression (42). Other chemokine receptors like CXCR1, CXCR4, CCR5, and CCR7 are infrequently expressed on mesothelioma cells isolated from pleural effusion of MPM patients (45). Therefore, those chemokine pathways might only play a role in a subset of MPM patients.

Activation of the colony stimulating factor-1 receptor (CSF-1R) through M-CSF or IL-34 can induce differentiation of monocytes to macrophages (46). Incubation of monocytes with pleural effusion from MPM patients or supernatant from MPM cell cultures resulted in a CD14^mid^CD163^high^ M2 immunosuppressive macrophage phenotype (47) (**Figure 2**). It has been shown that pleural effusions from MPM patients contain M-CSF and that they can induce differentiation of monocytes to M2-like macrophages in a CSF-1R dependent manner (48). M2 macrophages themselves can induce proliferation of MPM cells and induce treatment resistance to chemotherapies (47). Another study from Cioce et al. showed that autocrine CSF-1R signaling through AKT and β-catenin is a crucial signaling pathway for chemotherapy resistance and survival (49). Thus, the CSF1R/AKT axis represents an interesting target for further therapeutic development.

Other important factors for the differentiation of monocytes and macrophages like prostaglandin E2 (PGE2) and Transforming Growth Factor β (TGF-β) are present in pleural effusion and in the supernatant of MPM tumor cell lines (50, 51). PGE2 is an immunosuppressive factor in the TME and can induce a suppressive phenotype in macrophages with high suppressive capacity on T cell proliferation (52). The human monocytic cell line THP-1 activated with lipopolysaccharide (LPS), developed an immunosuppressive phenotype when co-cultured with the MPM cell line Mero84. An increased production of immunosuppressive cytokines like PGE2 and IL-10 was produced by macrophages with a shift towards M2 phenotype (53).

A very close interplay has been described between the production of TGF-β and priorities of macrophages in several settings. TGF-β is a critical cytokine in tissue homeostasis and can have pleiotropic functions in cancer. It can inhibit proliferation of cancer cells but also induce tumor progression and metastasis, thereby function as a tumor promoting cytokine (54). TGF-β concentrations in pleural effusions are significantly higher in MPM patients compared to those from primary lung cancer patients and they correlate with disease stage and tumor volume (55–58). Patients with high TGF-β concentrations in pleural effusions have significantly shorter survival, however, circulating serum TGF-β concentrations do not have a predictive value (58).

Upon production of the above mentioned chemokines, monocytes then become TAMs. TAMs have been widely described to express a pro-tumoral M2 phenotype, but recent studies suggest that they might have M1 and M2 properties at the same time (38, 59). M2 macrophages are considered to promote tumor growth, proliferation and invasiveness. Increased TAM levels correlate with poor survival, bad prognosis and increased metastasis potential in different tumors (60–63). In the human MPM tumor microenvironment, TAMs account for the majority of tumor infiltrating cells with about 25-40% of total immune infiltrates (23, 64). TAMs in MPM express an immunosuppressive M2 phenotype with high levels of the surface molecules CD163, CD206 and Interleukin 4 receptor α. Independent on the histological subtype, MPM is generally heavily infiltrated with macrophages without any correlation with tumor stage, but interestingly with survival in the non-epithelioid group (34) (**Table 1**). This could be due to presence of more immunosuppressive cytokines supporting the pro-tumoral role of infiltrated macrophages. A study by Marcq et al. showed a correlation between CD68^+^ macrophages with the presence of CD4^+^FoxP3^+^ regulatory T cells, accounting for a downregulation of the adaptive immune response and support of an immunosuppressive TME, which could explain partially the difference between the prognostic differences in survival (23). Nevertheless, the prognostic value of macrophages and in particular M2 macrophages has led to divergent conclusions, depending on the different studies and datasets, while certain studies report a significance others do not (16, 34, 65–67) (**Table 1**). In order to further dissect the role of these cells in MPM, an orthotopic mouse model of MPM was used, where a tumor promoting effect of macrophages was described: mice with a high tumor burden had higher numbers of macrophages/monocytes in the pleural effusion as well as higher percentages of M2 suppressive macrophages (71). In another orthotopic, syngenic murine peritoneal mesothelioma model, the tumor burden, measured by tumor growth rates, invasiveness and number of metastasis was significantly reduced when macrophages were depleted in these mice (72). Both studies indicate TAMs as a negative prognostic factor for tumor progression in mice.

**Table 1:** Summary of publications of overall survival correlated with immune infiltrates.

Number of cases	Histology	Cell Subset	Survival	Ref
667	Epithelioid and non-epithelioid group	Circulating monocytes	Negative correlation with overall survival	(34)
	Non-epithelioid group	CD68+ macrophages	Negative correlation with overall survival	
230	Epithelioid group	CD163+ macrophages	No correlation with overall survival	(39)
	Epithelioid group	CD163+/CD8+ ratio	Negative correlation with overall survival	
	Epithelioid group	CD68+ or CD163+ in stroma	Negative correlation with overall survival	
49	All histology, 75% epithelioid	M2 macrophages (CD68+, CD208+, Arginase-1+)	No correlation with overall survival	(65)
67	Epithelioid 49%, non-epithelioid 51%	CD68+ macrophages	No correlation with overall survival	(66)
8	Epithelioid group	CD163+/CD68+ ratio	Negatively correlated with overall survival	(67)
32	Epithelioid and non-epithelioid group	CD8+ T cells	Correlation with better survival	(68)
44	Epithelioid and non-epithelioid group	CD8+ T cells	Correlation with longer survival	(69)
302	Epithelioid group	CD4+ T cells	Correlation with better survival	(16)
	Non-epithelioid group	CD8+ T cells	Correlation with better survival	
	Epithelioid and non-epithelioid group	CD4+ FoxP3+ regulatory T cells	High expression is correlated with poorer survival	
93	Epithelioid and non-epithelioid group	CD4+ T cells	Correlation with better survival	(66)
	Epithelioid and non-epithelioid group	CD8+ T cells	Negatively correlated with overall survival	
302	Epithelioid group	CD20+	Correlation with better survival	(16)
93	Epithelioid group	CD20+	Correlation with better survival	(66)
230	Epithelioid group	CD20+	Correlation with better survival	(39)
88	Epithelioid and non-epithelioid group-PD-L1^+^	CD20+	Negatively correlated with survival	(70)

In conclusion, MPM seems to alter the myeloid cell differentiation program by tumor-derived factors, which contributes to tumor suppression and a pro-tumoral immune response. TAMs and monocytes could be a potential target to alter this effect and induce an anti-tumoral immune response.

### Myeloid-Derived Suppressor Cells

Myeloid-derived suppressor cells (MDSC) represent a pathological status of monocytes and neutrophils and are present in different pathological conditions. MDSC represent a small proportion of tumor infiltrating cells in MPM, below 10%, but with important pro-tumoral functions (65). MDSC promote tumor development and progression through different mechanisms, they have the ability to suppress T cells, remodel the TME, support EMT and angiogenesis (73, 74). MDSC are a heterogeneous group of myeloid cells but can be roughly divided in two groups, the granulocytic (Gr-MDSC) and the monocytic (M-MDSC) subset (74).

In MPM, the two populations of MDSCs can be found in the TME; the granulocytic like subtype expressing CD15^high^/CD33^low^ and the monocytic like subtype expressing CD15^low^/CD33^high^ (15, 67, 75, 76). Gr-MDSCs are recruited to the tumor site through G-CSF and GM-CSF released by MPM tumor cells and further differentiate into an immunosuppressive phenotype within the tumor (75, 77, 78). In MPM, MDSCs inhibit proliferation of CD8^+^ T cells through secretion of immunosuppressive molecules like reactive oxygen species (ROS), nitric oxide (NO) and kynurenine (75, 79, 80). High amounts of tumor infiltrating MDSCs significantly decrease progression free survival and overall survival in MPM patients (65).

Targeting MDSC in murine model of mesothelioma leads to reduced numbers of intra-tumoral MDSC with reduced capability to produce ROS and to reduce tumor growth (81). Targeting MDSC in MPM patients might represent a way to reduce intra-tumoral immune suppression and enhance immunotherapy regimes in future.

### T Cells

T cells play an important role in anti-cancer immunity in solid tumors and overall survival is closely linked to the presence of tumor infiltrating lymphocytes (TILs) across different tumor types (82). The approval of immune checkpoint inhibitors targeting CTLA-4 or the PD-1/PD-L1 interaction and their enormous clinical success in different malignancies (83, 84) further points out the importance of T cells in controlling cancer cells. In MPM CD3^+^ T cells are highly abundant in the TME and the presence of CD8^+^ TILs is a favorable marker for prognosis (68, 69). However, 60 to 80% of the cases analyzed are usually MPM tumors with an epithelioid subtype, and only few cases with the most aggressive sarcomatoid form are included. Sarcomatoid tumors show fewer CD4^+^ and CD8^+^ T cells in the tumor and are characterized by a loss of Th1 features such as T-bet (marker for Th1 polarization) and granzyme B expression, which are required for an efficacious anti-tumor immune response. In addition, the sarcomatoid subtype does not express HLA class I molecules leading to escape of T cell mediated cytotoxicity (17). Another study compared the epithelioid group with a non-epithelioid group (sarcomatoid and biphasic) and linked immune markers with outcome: high CD4^+^ counts in the epithelioid subset was associated with better prognosis, in contrary, in the non-epithelioid subsets, high CD8^+^ counts were associated with better prognosis, in both, high expression of FoxP3 was correlated to poorer survival (16, 66) (**Table 1**). Infiltration of T cells also varies between PD-L1 high and low expressing tumors. PD-L1 high tumors have more CD45^+^ cells infiltrated compared to PD-L1 low tumors, and significantly more CD3^+^ cells including CD4^+^ and CD8^+^ and regulatory T cells and express more co-inhibitory receptors such as TIM-3. Nevertheless, there is also a huge variability between patients, which could account for the differences in responses to immunotherapies (15, 85). Thus, the presence of TILs and the expression of PD-L1 are not sufficient to predict responses to such therapies, but rather the whole immune-context, including the presence of suppressive cells and inhibitory receptors might predict outcome. Other important factors for response to immunotherapy in MPM patients are the effector functions of TILs. On the one hand, cytotoxic T cells in MPM express more T cell immunoglobulin and ITIM domain (TIGIT) and TIM-3 compared to T cells from health lung tissue and had a minor ability to produce IFN-γ upon stimulation (86) (**Figure 2**). On the other hand, patients with MPM do have approximately double the amount of regulatory T cells (Tregs) cells in the periphery compared to healthy control. Tregs are important in sustaining peripheral tolerance and preventing autoimmune disease by suppressing other cells. The balance between effector T and B cells and Tregs is crucial for the quality and magnitude of the immune response. Nevertheless, the presence of Tregs can also block required anti-tumor immune responses (87) and their presence in tumors is associated with poorer prognosis in different malignancies (88). In a study from Klampatsa et al. around 12.8% of all CD4^+^ T cells in the MPM tissue are positive for FoxP3 compared to 2.2% from healthy lung tissue. In addition, there were significantly more Tregs in the tumor compared to the blood (86, 89). In a murine MPM model Tregs were also shown to be crucial for cancer progression, depleting Tregs with an anti-CD25 antibody led to reduced tumor growth (90).

## PD-L1 and Other Immune Checkpoint Inhibitors

Immune checkpoints have drawn attention during the last years due to the development of antibodies blocking the interaction of PD-1 and PD-L1 and its extraordinary success in cancer therapy. The PD-L1/PD-1 axis leads to inactivation of T cells. In the context of cancer, T cells are continuously exposed to tumor antigens, which leads to a state of dysfunctionality and unresponsiveness called exhaustion. Blocking the PD-L1/PD-1 axis with therapeutic antibodies reactivates T cells against cancer. PD-L1 is expressed in mesothelioma tumors, however, the positivity rate very much depends on the study, the cohort and the assay performed for analysis (23, 91–93). A recent overview analysis of four different antibodies used to stain for PD-L1 gave an incoherent picture over the different assays. The use of reliable antibodies and standardization of staining methods are important features in order to receive comparable studies (94). In MPM, high PD-L1 expression is associated with histology and is higher expressed in sarcomatoid/biphasic subtypes (66, 92, 95). In the recently published PROMISE-MESO trial, 48.9% of the patient had less than 1% PD-L1 expression, 28.2% had 1-20% and 18.5% more than 20% PD-L1 expression (96). Another study described 73% to be positive (> 1%) and 27% negative (<1%) (97). Inaguma et al. describe 33% of MPM to be positive and 67% negative. In addition, high PD-L1 expression is negatively correlated with overall survival (85, 95, 98). Importantly, PD-L1 expression is not only restricted to TME but also on tumor cells of the pleural effusions (99); therefore, further investigations with detailed analysis of abundance and localization of expression are warranted.

Recent studies have identified a wide range of other immune checkpoint molecules beside PD1/PD-L1, which could be suitable for cancer treatment, in particular TIM-3, LAG-3, TIGIT and V-domain Ig suppressor of T cell activation (VISTA). TIM-3 plays a major role in controlling the function of NK and T cells. Upregulation of TIM-3 on peripheral immune cells and its cognate ligand galectin-9 on tumor cells inhibits immune responses. Galectin-9 is expressed on MPM tumors where it can suppress T cell response (65). Furthermore, a higher number of TIM-3^+^ cells in peripheral NK and T cell populations correlate with a poor prognosis in many solid tumor types (20). In MPM, T cells in pleural effusion express inhibitory molecules such as PD-1, TIM-3, LAG-3 and have a higher diversity of TCR clones compared to blood of the same patient (65, 100). Interestingly, the *LAG-3* gene is higher expressed on mesothelioma tumors compared to lung adenocarcinoma, while *PD-L1* gene is higher expressed is lung adenocarcinoma (101). Understanding differences in the TME of various solid tumors can open up new options for more personalized immunotherapeutic approaches. A recent study defines a subgroup of patients co-expressing inhibitory molecules TIM-3, PD-L1, CTLA-4 and LAG-3 where this expression is associated with a shorter survival (102). Another immune checkpoint molecule, VISTA, is a negative regulator of T cell activation and it is highly expressed on myeloid cells. Similar to PD-L1, VISTA can support the conversion of naïve T cells to FoxP3^+^ regulatory T cells (103, 104). In MPM, VISTA is highly expressed on the epithelioid subtype but to a lesser extent on more aggressive subtypes. Interestingly, it is also expressed on normal and reactive mesothelium (105). In contrary to PD-L1 patients with high VISTA expression had a better overall survival, moreover, patients with concurrent high expression of VISTA and VEGFR2 survive almost five times longer compared to patients with low expression (102, 106).

## B cells

B cells are essential cells of the adaptive immune system and function as antigen presenting cells (APC) thereby contributing to T cell activation, differentiation and polarization. B cells also play an important role in promoting the formation of tumor-associated tertiary lymphoid structures (TLS), areas for B cell maturation and isotype switching (107). The presence of B cells in the tumor can be a prognostic factor in different malignancies (108). In MPM, only few studies addressed the function and importance of B cells in the tumor and in pleural effusion. Krishnan et al. showed high levels of tumor-specific antibodies in murine models of mesothelioma treated with immunotherapy compared with untreated controls. In addition, disease eradication in all treated animals and complete failure of the treatment in B cell-deficient mice have been demonstrated (109). These findings line up with previous preclinical data, which showed increased levels of IgM and IgG after anti-CD40 antibody treatment and during tumor regression in mice (110). This data suggests that antibodies generated upon treatment play an important role in the tumor immunity and are essential for tumor responses (**Figure 2**). Besides high levels of antibodies, Jackman et al. showed an increased percent of B cells in the tumor as well as in the secondary lymphoid organs. In contrast, the number of T cells and of the other cells of the immune system (e.g., macrophages, NK, granulocytes) remained low (110). In a previous study, high antibody titers against four tumor-associated antigens (GeneX, THBS-2, STUB-1 and IFT88) were identified in the *sera* of MPM patients. In particular, high levels of antibodies against two of those antigens, GeneX and THBS-2, were detected in almost all MPM patients, with a decrease after surgical resection (111). These findings represent another example of the existence of a specific humoral immune response in MPM patients: antibodies produced by tumor infiltrating B cells can be used as a tumor marker for diagnosis and follow up. The association of high B cell numbers in the tumor with survival has been recently described. Different studies showed that high counts of CD20^+^ cells in patients with epithelioid mesothelioma positively correlated with survival, however, this was not the case in the non-epithelioid subgroup (16, 39, 66) (**Table 1**). In contrast, another study identified elevated B cell numbers in the sarcomatoid subgroup (70). In addition, in PD-L1^neg^ MPMs B cells are considered a good prognostic factor, whereas in PD-L1^+^ MPMs, CD20^+^ infiltrates are associated with a poorer outcome (70) (**Table 1**). Patil et al. classified MPM cases into three different subgroups, based on immune profiles: one subgroup showed higher expression of B cell markers and antigen presentation-related genes compared with the other ones (91). This indicates that B cell infiltration is not a constant feature in all MPM cases and cannot be considered as a hallmark as, sometimes it is rarely detectable (76). Nevertheless, in some patients increased levels of B cells constitute a window of opportunity to develop novel immunotherapies and to identify novel MPM targeting receptors. Through bulk RNA-Seq data analysis from MPM tissue, the BCR sequence can be identified to generate candidate antibodies binding to MPM target cells. Another new approach to produce high-affinity antibodies involves the isolation of memory B cells from peripheral blood of the donors, when immortalized, such B cells stably secrete monoclonal antigen-specific antibodies, which could be used as further therapeutic agents (112). More research is needed to better understand the role of B cells in MPM but based on these premises, this cell population can be considered an important candidate for the development of new therapies.

## Genetic Alterations Predisposing to Mesothelioma

As previously described, the role of external agents in inducing mesothelioma is mainly attributable to the chronic inflammation guided by *HMGB1*, NF-kB and the PIK3CA pathway. Meanwhile, clinical studies of large cohorts of individuals and the improvement of genome wide sequencing technologies have helped the identification of an increased number of oncological diseases associated with germline mutations (113). These genes mainly encode for tumor suppressor proteins involved in cell cycle regulation, apoptosis and DNA repair pathways; being involved in cancer development, they are called cancer susceptibility genes (113). The identification of these genes have paved the way for the development of targeted therapeutic approaches as well as for cancer prevention and surveillance (114, 115). The most common inherited cancer risk factor associated with MPM is the aberration of BRCA1 Associated Protein 1 (*BAP1*), a deubiquitinating enzyme located on chromosome *3p21.1* acting as tumor suppressor gene (116, 117). *BAP1* is involved in different biological pathways, such as DNA replication, apoptosis, regulation of gene transcription, deubiquitation of histones and DNA repair (116, 117). Germline defects of *BAP1* are responsible for the BAP1-tumor predisposition syndrome (BAP1-TPDS) including the occurrence of renal cell carcinoma, uveal melanoma, cholangiocarcinoma and mesothelioma. High frequency of germline mutations of *BAP1* were demonstrated to cause mesothelioma in 2001, when an epidemic spread of cases was reported in a village in Cappadocia (113, 118). These results were further confirmed by other groups: in 2018, Betti et al. reported a frequency of 7.7% of pathogenic germline variants in a cohort of 39 patients (119), while Pastorino et al. reported a frequency of 30.7% in a cohort of 52 patients affected by malignant mesothelioma (120).

*BAP1* alterations occur in one mutant allele and are inherited as autosomal dominant mutations: a study of germline-mutated mesothelioma showed 43.1% of relatives carried the same mutation of their probands (120). The peculiarity of mesotheliomas carrying *BAP1* defects is a higher frequency in non- or low-exposure to asbestos as reported by Pastorino et al (120). These alterations are more frequently detected in young adults with MPM: 4% in patients older than 75 years and 20% in the ones of 55 years of age or younger (121). Among the three histotypes of MPM, such as epithelioid, sarcomatoid, and biphasic, BAP1-germline mutations are found more frequently in the epithelioid and this correlates with a better prognosis (113, 115).

Today, the assessment of *BAP1* status has become part of the diagnostic routine of mesothelioma allowing to distinguish between benign and malignant mesothelial cells and to identify biphasic mesothelioma (116). However, the implementation of sequencing technologies with more extensive studies will uncover molecular features associated with mesothelioma onset. Interestingly, a recent study has revealed that *BAP1* is not the only cancer-susceptibility-gene predisposing to mesothelioma. Bononi et al. have shown that heterozygous mutations in the Bloom syndrome gene (*BLM*), a gene involved in the DNA repair, promote the development of mesothelioma and the risk is further increased by exposure to asbestos (122).

These results suggest the importance of early detection of cancer risk factors in the population *via* tailored screening programs in order to prevent cancer development.

## The Role of the DNA Repair

The DNA damage response (DDR) consists of a complex network of genes that respond to different types of DNA damages, such as Double Strand Breaks (DSBs) and Single Strand Breaks (SSBs), being organized in pathways as homologous recombination (HR), non-homologous end-joining (NHEJ), mismatch repair (MMR) and nucleotide excision repair (NER). Defects in one or more pathways lead to genomic instability and promote tumorigenesis and cancer progression (123, 124).

DDR has been an attractive therapeutic target upon the discovery of synthetic lethality, which occurs when the inefficiency of a DNA repair system causes the recruitment of other DNA damage pathways. This concept has first been applied to HR genes like *BRCA1/2* due to their interaction with the poly(ADP-ribose) polymerase (PARP), a family of proteins that are activated in the presence of DNA damage and stabilize the replication machinery during repair (123). Thus, a new category of drugs called PARP-inhibitors (PARPi) have been developed to target the rescue of DNA repair pathway and lead to genomic instability and cell death (123). Among the most studied PARPi there are talazoparib, rucaparib, niraparib, olaparib and veliparib; although being first intended to target tumor lacking functional *BRCA1/2*, their action has been expanded to a larger category of Homologous Recombination Deficiency (HRD) tumors, that comprise other HR genes like *ATM*, *ATR*, *RAD51*, *BARD1 (* 124).

Evidence reported in several works has shown a high percentage of germline mutations in MPM occurring in DNA repairing genes. Betti et al. tested a panel of 94 cancer predisposing genes and found mutations in *PALB2*, *BRCA1/2*, *FANC1*, *ATM*, *SLX4*, *FANCC*, *FANCF*, *PMS1* and *XPC* covering almost the 10% of all tested patients (118). Most of these genes were involved in specific DNA repair mechanisms like HR, MMR and NER. A similar result was further confirmed by Panou et al. that reported an improved survival in patients with MPM bearing DNA repairing defects (121). A study by Guo et al. addressed the role of DNA repair genes in the pathogenesis of MPM and identified mutations in novel target genes like *MSH3*, *BARD1* and *RECQL4* that have not been previously described (125). A recent review by Fuso Nerini et al. confirmed that considering different studies performed on MPM, the DNA repair pathways are among the most frequently affected (124).

In this context, the use of PARPi in mesothelioma has been encouraged, however preliminary data do not allow a clear conclusion of PARPi effectiveness (124) (**Table 2**). In fact, while some studies have shown combinatorial treatment with cisplatin and olaparib is effective in mesothelioma cells with a defective HR (137), another study has demonstrated that olaparib has limited anti-tumor activity also in *BAP1* mutated patients (NCT03531840). Other clinical trials are still ongoing (NCT03207347, NCT03654833) and will help clarify the effects of PARPi in mesothelioma.

**Table 2:** Druggable targets in mesothelioma.

Molecular Feature	Drug	References
BAP1	PARPi (olaparib, niraparib, rucaparib)	NCT03531840, NCT03207347, NCT03654833
MGMT low, SFLN11 high	PARPi (talazoparib) + temozolomide	(126)
ALK fusion	ALK inhibitors	(127)
BAP1 *wt* and KDM6A	tazemetostat	(128)
NF2	FAK inhibitors, everolimus	(129, 130)
PTCH1	vismogedib	(131)
TERT	telomerase inhibitors (MST-312)	(132)
DNA repair and TME	lurbinectedin	(133)
BCL2, BCL-XL	BH3-mimetics, survivin inhibitor(YM155), bortezomib, trabectedin	(134)
HDAC	vorinostat	(135)
STAT1	fludarabine (F-araA), risedronic acid (RIS)	(136)

One of the main concern about DNA damage and mesothelioma has been the assessment of the role of *BAP1* in sensing cells to PARPi. The role of *BAP1* in the DDR is due to its interaction with *BRCA1* and *BARD1*, however this association is far to be completely understood and need further investigation (116). A recent study exploited the sensitivity to PARPi in patient-derived mesothelioma cells (126). They showed that response to PARPi is independent on *BAP1* mutational status. Conversely, they demonstrated that PARPi sensitivity, especially to talazoparib in combination with temozolomide, is mainly related to the combination of low expression levels of O-6-Methylguanine-DNA methyltransferase (*MGMT*) and high expression levels of Schlafen 11 (*SLFN11*) (126) (**Table 2**). Overall, this evidence suggests that targeting the DDR in MPM is still an attractive strategy, most of all in a context of combined therapy; however, more preclinical studies are needed to exploit other combinations and unravel molecular mechanisms and drug-interactions that could lead to improved patients outcome.

## Therapeutic Implications of Genomics and Transcriptomics Evidences

The *-omics* field in the study of cancer pathology has evolved rapidly in the last decades. The improvement of high-throughput technologies and computational approaches have made a big step forward in cancer characterization and drug-response investigation becoming crucial in the context of translational research.

Genomic and transcriptomic studies have improved the molecular characterization of MPM and set new hypothesis for therapeutic approaches. The first genomic studies from Bueno et al. in 2016 analyzed 211 transcriptomes and 216 whole exomes of mesotheliomas, while Hmeljak et al. in 2018 analyzed 74 samples from The Cancer Genome Atlas (TCGA) by the integration of the exome and the transcriptome (105, 138). Both studies confirmed frequent mutations in the *CDKN2A*, *NF2*, *TP53*, *LATS2*, and *SETD2* genes and a low mutational burden with less than two non-synonymous mutations per megabase (Mb). Bueno et al. identified other genomic aberrations such as gene fusions and splice alterations in the most relevant genes like *NF2*, *BAP1* and *SETD2 (* 138 *).* Both studies assessed a somatic copy-number alteration (SCNA) landscape with more copy losses than amplifications that included *BAP1*, *NF2*, *CDKN2B*, *LATS2*, as a further confirmation that MPM development is driven primarily by loss of tumor suppressor genes than by activation of classic oncogenic drivers (105, 138).

The pivotal role of *BAP1* in mesothelioma is confirmed also at the somatic level, since 60% of cases present a second hit (120), even if the percentage could be even higher since studies performed so far have used next generation sequencing (NGS) approaches that lacked the identification of large deletions, while assessment from different platforms, like IHC and multiplex ligation–dependent probe amplification (MLPA) have increased detection perfomances (116). Somatic *BAP1* mutations preferentially affect the epithelioid subtype and correlate to better prognosis (116).

Recently, a work by Zhang et al. has depicted a detailed picture of MPM genomic features. Indeed, the study of intratumor heterogeneity of MPM through an exome sequencing approach has shown that most MPMs follow a linear evolution with BAP1 being the most frequent ancestral mutation and NF2 arising mainly as a late event. Moreover, a minority of patients presented a branched evolution that was associated with a higher tumor lymphocyte infiltration and antigen burden, suggesting a possible sensitivity to immunotherapy (139).

Aberrant copy number alterations in *CDKN2A* and *p16* arm identified with sequencing approaches were confirmed in other studies through fluorescent *in-situ* hybridization (FISH) and IHC and they were associated with higher asbestos fiber exposure (140).

Somatic mutations in *CDKN2A*, *NF2*, *BAP1* were also reported in cases of malignant peritoneal mesothelioma, with *CDKN2A* less frequent as compared to pleural mesothelioma (141). Interestingly, the same work reported gene fusions such as EWSR1-ATF1 and FUS-ATF1 and *ALK* rearrangements that are hardly found in pleural mesothelioma and seem to be specific for young women as compared to *ALK*-*wild type* patients but might respond to targeted treatment (127, 141, 142) (**Table 2**). Two recent studies have reported novel somatic mutations in *RDX* and *MXRA5* genes, independently (143, 144). In Torricelli et al. *RDX* and *MXRA5* are present in 42% and 40.6% of the cohort, respectively and authors stated that *MXRA5* is specific for the biphasic histotype together with *NOD2 (* 143). The same genes were also described in the RAMES study where *RDX* and *MXRA5* represented the 42% and 23% of MPMs, respectively, however in this case *MXRA5* was identified in both epithelioid and non-epithelioid histotypes (144). Moreover, the same gene is reported to be significantly correlated to longer survival rate in a cohort of epithelioid only MPMs (145). Therefore, although the correlation of *MXRA5* to histopathologic or clinical features needs further interpretation, more studies on this gene and *RDX* are warranted.

The extensive work of the genomic studies presented so far have defined various genetic features of mesothelioma, but, to date, they have no role in patient stratification and treatment. Interestingly, more insights in MPM molecular characterization have emerged from transcriptomic studies. The first molecular classification in epithelioid, sarcomatoid, and biphasic, was proposed in 2016 with the identification of 400 most variable genes within the groups, 189 upregulated and 241 downregulated, which also correlated with survival (138). Specifically, the epithelioid subtype presented up-regulation of *UPK3B*, *ELMO3, CLDN15* while *LOXL2* and *VIM* were up-regulated in the sarcomatoid subtype, thus showing a key difference in EMT regulation in the two groups. Using a data-integration approach Hmeljak et al. came to the same histotype classification, however, they stressed a relevant issue: since MPM transcriptomic can be used for histotype stratification, it is possible to use it even further for prognosis within a single histotype. For example, although the epithelioid histotype has a better survival rate than sarcomatoid, even within this subgroup it is possible to identify different clinical courses. Guided from this hypothesis, a cluster of patients with epithelioid subtype with poor prognosis was identified and associated with higher *AURKA* mRNA expression (105). Previously, the association of *AURKA* expression to a worse prognosis was detected by Borchert et al (137). This evidence suggests that for a further improvement in MPM therapeutic approaches it is essential to consider integrated data analysis, such as the combination of genomic and transcriptomic features.

## Extensive Chromosomal Breakage: A New Identified Feature in Mesothelioma

**Figure 3:**
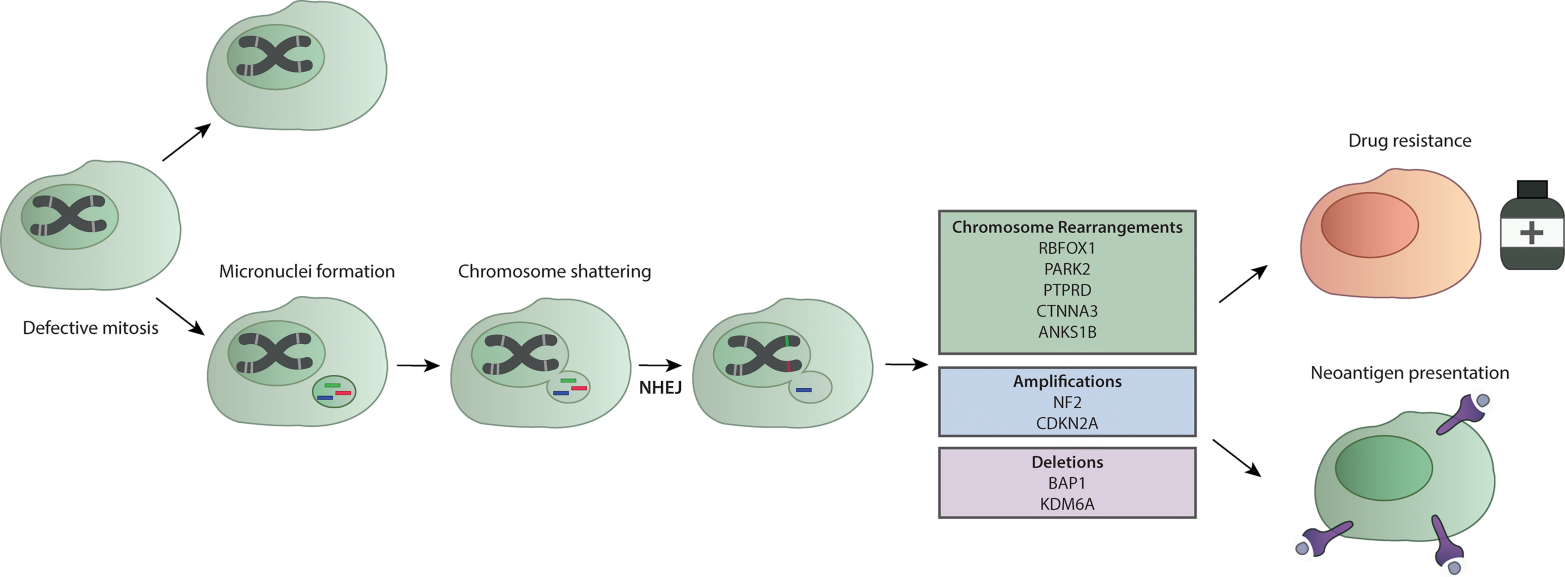
Chromotripsis in mesothelioma. A defective mitosis process leads to the formation of micronuclei. Further replication leads to chromosome shattering and the addition of genetic material through the NHEJ. This process forms chromosome rearrangements that in MPM interest genes like *RBFOX1, PARK2, PTPRD, CTNNA3* and *ANK51B*, and are linked to amplification of key MPM genes like *NF2* and *CDKN2A*, or the loss of genetic material of *BAP1* and *KDM6A* (113, 150, 151). Amplification of oncogenes due to chromotripsis has been recently associated with drug resistance (152). The extensive chromosomal breakage upon chromotripsis events leads to neoantigen presentation that can make cells more sensitive to immunotherapy (150).

As previously stated, mesothelioma presents as a tumor with low mutational burden with a median of 23 mutations per biopsy specimen (146) with ~1.2 mutations per Mb (105, 138). This finding identifies mesothelioma as an atypical tumor, since it is known that exposure to carcinogens and environmental pollution characterize tumors with a highly compromised genomic landscape and high mutational burden. However, latest development of sequencing technologies have revealed hidden aspects in cancer malignancies that have not been previously investigated, such as chromotripsis. The word chromotripsis derives from “chromo” which stands for chromosome and “tripsi” which means breaking into small pieces (147) and refers to a mutational phenomenon of DNA breakage from a single event that spreads into hundreds of catastrophic chromosomal damages (148). Accumulated DNA damages lead to the formation of micronuclei, usually containing single chromosome, that are disrupted during cell cycle and spread genetic material in the cells (146). Pieces of chromosomes can be included in the nuclei during mitosis, and this generates chromosome rearrangements and fusions (149) (**Figure 3**). Recent studies have addressed this topic in malignant mesothelioma. With the intent to provide reliable cell line models of mesothelioma, Oey et al. have characterized the genome of tumors and tumor-derived cell lines through whole genome sequencing (151). Here, the authors have identified recurrent events of high chromosomal instability like chromoanagenesis and chromotripsis. Interestingly, inter- and intra-chromosomal rearrangements affected genes like *CDKN2A*, one of the most frequently mutated in mesothelioma and *KDM6A*, a gene that has been associated with sensitivity to enhancer of zeste homolog 2 (EZH2) inhibitors, like tazemetostat (151, 153). This finding might have important implications, as *BAP1*-lacking mesotheliomas have demonstrated sensitivity to EZH2 inhibitors (128) (**Table 2**).

A more recent study addressed chromotripsis in mesothelioma through a new approach (150) called Mate-pair sequencing (Mpseq). Mpseq generates larger sequencing fragments and can detect chromosomal rearrangements and large insertion/deletion (150). Mansfield et al. found rearrangements that lead to *CDKN2A, BAP1* and *NF2* chromosomal instabilities. In particular, they have evaluated the MESO cohort of TCGA and identified chromotripsis events in 69% of patients, mostly occurring in tumor suppressor genes and non-coding DNA (150).

Results obtained in other cancers have shown that chromotripsis contributes to oncogene amplification, thus promoting cancer progression (152, 154). Among them, those harboring telomerase reverse transcriptase (*TERT*) gains have shown higher prevalence for chromothripsis (149) and this can be linked to mesothelioma with TERT-impairment that present poorer prognosis. This evidence suggests that chromotripsis is a specific genomic feature of mesothelioma and that future studies should include investigations of this complex event, especially for therapeutic developments. To this, chromotripsis has been recently associated with the development of drug-resistance in cancer through a mechanism that involves the repairing systems of the DNA *via* PARP and the NHEJ pathway (152). In addition, the notion of mesothelioma as a low mutated tumor should be reconsidered since most studies have defined the mutational burden in relation to only nucleotide changes, however the previously discussed findings suggest to consider genomic and structural rearrangements as well. Indeed, an increased expression of neoantigens resulting from these catastrophic events in mesothelioma correlate with clonal expansion of tumor-infiltrating T cells (150) suggesting a possible role in response to immunotherapy (**Figure 3**). All together, these discoveries indicate that the group of MPM presenting chromotripsis could benefit from combinatorial drug treatment, including PARPi or immunotherapy, that are worth being further exploited.

## Novel Molecular Targets for Therapeutic Strategies

Following *BAP1*, the second most frequent mutated gene in mesothelioma is the neurofibromatosis type 2 (*NF2*) which encodes the protein merlin (138). This protein plays a role in the Hippo and the mTOR pathways, other than being involved in EMT (155). In mesothelioma, *NF2* is found mostly as a biallelic inactivation. As reported by Sato et al. preclinical *in vivo* studies have shown the central role of *NF2* in sensitizing tissue to asbestos and developing mesothelioma (129). In other studies, conducted on patients cohort, it has emerged that impairment of the *NF2* gene is more frequent in the sarcomatoid subtype rather than the epithelioid and this correlates with worst prognosis (129, 138). To date, NF2 alterations represent a possible target for treatment such as drugs aiming at interfering with its function in extracellular matrix signal transduction. Up to date, studies are mainly based on preclinical models, cell lines or xenograft; however, promising results suggest further developments. To this, the initial studies investigating FAK inhibitors as VS-4718, or YAP and mTOR inhibitors in MPM lead to positive results in preclinical studies but not in clinical trials (129) (**Table 2**). Others showed the antagonistic relationship between FAK and Wnt pathways in malignant mesothelioma: dysregulated Wnt signaling is associated with invasion and resistance to apoptosis, while FAK signaling promotes invasion and EMT (130). The most interesting outcome has been reached with the development of K-975, a small molecule that inhibits the transcriptional enhanced associate domain (TEAD) protein that belongs to the Hippo pathway. K-975 showed a potent inhibitory effect on the proliferation of MPM cell lines, with a greater activity on NF2-non-expressing cells (156). Overall, these studies suggest that *NF2* could be considered as a potential target for MPM warranting further investigations.

Recently, the involvement of the Hedgehog pathway in MPM has been investigated. Here, in a patient diagnosed with epithelioid MPM, a mutation in *PTCH1* was identified (131). This gene is involved in the Hedgehog pathway and is druggable by vismodegib, already approved for the treatment of basal cell carcinoma (BCC) (**Table 2**). Despite the patient underwent several lines of treatment, vismodegib led to a very good partial response which lasted for over two years. The lack of genomic testing throughout the course of chemotherapy does not allow understanding whether the *PTCH1* mutation was acquired under pharmacological pressure; still, this result suggests an interesting novel target in MPM.

Pirker et al. investigated the role of *TERT* promoter in mesothelioma (132). They found that mutations in *TERT* are prevalently associated with non-epithelioid subtype and to poor survival. In accordance with this evidence, *TERT* mutations are mutually exclusive with *BAP1*, which is more frequent in the epithelioid subtype and correlates with better prognosis, as previously discussed. An interesting aspect is that cell lines derived from tumors with mutated *TERT* present a more stable genome, e.g., in the number of gains or losses, in comparison to wild type ones, with a specific imbalance in chromosomes 1, 5q, 9p, 7, 14, and 20. These evidences brought to test telomerase inhibitors like MST-312 MPM, however with moderate effects (132) (**Table 2**).

BCL-2 is a family protein involved in tumorigenesis of different cancer types. It consists of BCL-2, BCL-XL, BCL-W, MCL-1 and BFL-1 proteins. In mesothelioma, overexpression of *BCL-2* was found in 20% of cell lines and in 24% of a tumor samples, while the expression of *BCL-XL* and *MCL-1* was identified as a general feature of mesothelioma, suggesting their critical role as pro-survival factors (134). As BCL-2 proteins act directly on the apoptosis pathway downstream from *TP53*, this makes its targeting an interesting therapeutic approach (134). Preclinical studies have been performed in mesothelioma cell lines using direct or indirect targeting of BCL2 and BCL-XL: both in cell lines and *in vivo* models this treatment was able to induce an apoptotic effect. This has paved the way to the development of BH3-mimetics a novel class of compound that mimic the interaction of BCL-2 protein through their BH3 domain. In mesothelioma, BH3-mimetics have been used in combination with YM155, a survivin inhibitor, or bortezomib, with an increased apoptotic effect in comparison to controls (**Table 2**). These drugs have also been used in combination with trabectedin, a marine compound, showing that *BCL-2* mRNA expression inversely correlated with response to the treatment (134).

The marine-derived drug trabectedin has been already tested in MPM, although without any efficacy. In fact, the ATREUS trial, where this drug was administered as second line therapy in epithelioid MPM and as a first or second line in non-epithelioid subtype, showed poor efficacy of trabectedin and high liver toxicity that did not justify further use of this drug (157). However, lurbinectedin, an analogue of trabectedin, has shown promising results in a phase II clinical trial, where it was administered as a second or third line therapy in MPM patients (133) (**Table 2**). Lurbinectedin was efficacious independently on the MPM histotype and previous treatment. Further investigations to understand which patients might benefit from this drug are needed; however, the possible dual effect of lurbinectedin on both tumor cells and microenvironment might open new therapeutic venues for this disease (158).

In a recent work Dell’Anno et al. have used a drug repositioning approach to screen five MPM cell lines with 1170 FDA-approved drugs (136). They identified fludarabine (F-araA) and risedronic acid (RIS) as effective in MPM through a mechanism of inhibition of STAT1 expression and nucleic acids synthesis. Although promising, these results are yet limited by the use of only five cell lines without molecular characterization of these cells. Further studies including diverse and deeply characterized models are needed to successfully develop treatments for MPM.

## The Role of MicroRNA Dysregulation

In the past 20 years it has become increasingly clear that microRNAs are important players in the regulation of physiological processes within the cells, and that their dysregulation can be a major driver of malignant transformation and tumor progression (159, 160). Following the first description of a direct link between the loss of expression of a specific microRNA and cancer in 2002 (161), many studies followed investigating both the oncogenic and tumor suppressive potential of microRNAs. Hence, to date we know of many microRNAs that are involved in several processes linked to cancer development and progression, from regulation of cell proliferation and cell cycle processes to EMT and involvement in cancer immune escape (160, 162–164).

While still somewhat understudied in MPM, the studies available thus far, have shown that microRNAs play an important role in the biology of MPM, and represent valuable biomarker candidates (165, 166).

Considering that MPM is a diseases characterized by the loss of tumor suppressors, it is not surprising that this is also reflected on microRNA level with the majority of dysregulated microRNAs being lost, while only a limited number has been shown to be upregulated in MPM (165–167). The first study investigating dysregulation of microRNAs in MPM was performed by Guled et al. in 2009, who could show differential expression of microRNAs in MPM tissue compared to normal pericardial mesothelium, but also differential expression between the different histological subtypes of MPM (168). This study was shortly after followed by a study from Busacca et al. on MPM cell lines compared to immortalized mesothelial cells, which showed that the microRNAs with the greatest differential expression between MPM and normal cells, also discriminated between histopathological subtypes when investigated in tumor samples (169).

While the dysregulated microRNAs identified in these early studies were not functionally investigated, the obtained data strongly hinted towards a relevant role for microRNAs in MPM biology. The first studies providing functional data on dysregulated microRNAs, investigated the effect of re-expression of miR-29c-5p (miR-29c*) (170) and miR-31 (171), and in both cases, re-expression using microRNA mimics resulted in reduced proliferation and invasion of MPM cell lines, supporting a tumor suppressive role of these microRNAs. Following these initial studies, further tumor suppressor microRNAs were investigated in MPM such as miR-145 (172) and miR-205 (173), both of which are likely to alter EMT *via* targeting OCT4 and ZEB1/2 respectively. One of most comprehensively investigated tumor suppressive microRNAs in MPM is probably the miR-15~107 super family, and here in particular the family member miR-16. Reid et al. have shown that the members of this microRNA superfamily are quite consistently lost in MPM tissue compared to normal pleura, as well as in MPM cell lines (174). *In vitro* analyses then revealed that re-expression of miR-16-5p resulted in reduced cell proliferation and colony forming ability, as well as induction of apoptosis and a G0/G1 cell cycle arrest in MPM cell lines, but not in the non-malignant mesothelial cell line MeT-5A. These observed effects were most likely brought about by the downregulation of miR-16-5p target genes *BCL2* and *CCND1*. In addition, re-expression of miR-16 resulted in sensitization of cells to pemetrexed and gemcitabine, suggesting an additional role of the miR-15~107 family in response to antimetabolite chemotherapy. Most importantly, mouse experiments, in which miR-16 was systemically delivered to the tumor cells using (EnGenic) minicells resulted in significant tumor growth inhibition *in vivo*. Based on these data, a phase I clinical trial was performed in MPM and non-small cell lung cancer (NSCLC) patients, which apart from reaching its goal of proving safety of the miR-16 replacement therapy approach also showed in one patient a remarkable metabolic and radiological response to the treatment (175, 176). In a more recent study, the same group has now shown that miR-16-5p is a regulator of PD-L1 expression, hence also linking this tumor suppressor to response to immune checkpoint inhibition (177). While miR-16-5p is the most comprehensively investigated tumor, suppressive microRNA linked to MPM biology, other microRNAs such as miR-193a-3p (178), miR-137-5p (179), miR-126 (180, 181), miR-34b/c (182, 183), and miR-215-5p (184) have also promising anti-proliferative and anti-tumor activity, when re-expressed *in vitro* or *in vivo*.

Compared to the tumor suppressive microRNAs, which have been investigated relatively frequently in MPM, studies of oncogenic microRNAs are much rarer, also due to the fact that not many microRNAs have been found to be consistently overexpressed in MPM tumor tissue. One example however are miR-182-5p and miR-183-5p, which inhibition using microRNA inhibitors results in reduced proliferation and invasion (185).

Taken together, the available expression and functional studies highlight that microRNA dysregulation, in particular the loss of tumor suppressive microRNAs, is likely to represent an important contributor to MPM biology, and therefore to the development and progression of this devastating disease. Considering the encouraging data obtained from *in vivo* microRNAs replacement studies and early clinical trials, additional research efforts in this area are certainly warranted. Especially also in light of the recent development of mRNA-based vaccines against COVID-19, the field of RNA-based vaccines has significantly advanced, possibly also opening additional avenues for delivery of microRNAs to cancer cells in order to replace lost tumor suppressors.

In addition to their important role in contributing to MPM biology, microRNAs have in recent years also been investigated for their biomarker potential both in tumor tissue and in blood (165, 166). Regarding the diagnostic value of tumor microRNA expression, the first published study by Gee et al. performed microRNA profiling in MPM tissue and pleural metastases from lung adenocarcinomas (186). This study identified in particular low expression of members of the miR-220 family as potential diagnostic factors for differentiating MPM and pleural metastases. A subsequent study by Benjamin et al., then investigated microRNA expression in a larger set of MPM and adenocarcinomas of the lung or pleura, and identified in addition to members of the miR-200 family, also members of the miR-192 family as potential diagnostic markers (187). Based on this data, a microRNAs expression signature was generated and independently validated in a small set of patients. The signature consisting of miR-192, -193-5p and -200c showed high accuracy (95%) in discriminating MPM from adenocarcinomas, and was subsequently marketed by Rosetta Genomics. While additional candidates such as miR-126, -143, -145 and -652 have been proposed (188, 189), none of the microRNAs or diagnostic signatures described here has been followed up extensively, hence none of them is in routine clinical use.

Similarly, several studies have been undertaken investigating the prognostic value of microRNAs in MPM. Among the most promising candidates is the tumor suppressive microRNA miR-29c-5p, which expression is not only associated with the histological subtype (higher in epithelioid), but also significantly associated with survival (170). Similarly, the tumor suppressor miR-31 has recently also been proposed to hold prognostic potential, with lower expression being associated with longer survival (190). Furthermore, in a study investigating specimens from patients undergoing extrapleural pneumonectomy, a signature consisting of 6 microRNAs, the so-called miR-Score, was identified, which was able to separate patients with good and poor prognosis with an accuracy of 87% (191). However, similar to the diagnostic candidates, independent validation studies are lacking.

An attractive alternative to microRNA expression in tissue samples is the presence of microRNAs in the circulation. Other than longer RNA species, microRNAs show remarkable stability in the blood of patients due to the fact that they are mainly bound to lipoproteins or encapsulated into extracellular vesicles. With this in mind, a small number of studies has been published proposing for example low levels of miR-103 in whole blood (192, 193), low levels of miR-126 (181, 188, 194) in serum, low level of miR-132 in plasma (195) or high levels of miR-625-3p (196) in plasma or serum to be associated with the presence of MPM, and allowed to discriminate those patients from healthy controls and asbestos-exposed individuals. While these studies again provide first evidence of the biomarker potential of microRNAs in the circulation, large independent validation studies undertaken by independent research groups are usually missing, and none of the candidates is yet ready for routine clinical application.

## Epigenetic Alterations as Promising Prognostic Biomarkers

In addition to the above mentioned specific alterations of DNA or RNA in MPM, great interest is now being paid to the epigenome, that includes post-translational modifications that ultimately impact on gene expression not encoded by the DNA (197). Epigenetics modifications play an important role in the regulation of gene expression and include DNA methylation, histone modifications and chromatin remodeling. Dysfunction of these mechanism have been linked to tumorigenesis, cancer progression and metastasis (198, 199).

Due to the lack of druggable molecular targets, the level of investigation in MPM disease has moved so far. DNA methylation associated with tumor-suppressor genes and mechanisms involving histone modifications have been described and linked to MPM phenotypes and histological subtypes (198). DNA methylation provokes gene silencing by adding a methyl group to the fifth carbon of the cytosine base, with a process that mainly occurs in promoter regions, thus modifying the expression of the associated genes, or on repetitive DNA elements, such as LINE1, causing chromatin modifications (200). Although DNA methylation patterns are fairly stable markers of differentiated tissues that regulate specific gene expression, changes in the methylation profile can occur due to aging, exposure to environmental stimuli and chronic inflammation (200, 201). In particular, accumulation levels of DNA methylation has been associated with higher cancer risk and cancer onset (202). The investigation of the DNA methylation profiles can be carried on at the tumor level, otherwise it is possible to search specific markers in the circulating peripheral blood. Studies conducted in malignant mesothelioma tumors have identified hypermethylated regions in *TMEM30B*, *KAZALD1* and *MAPK13 (* 203). DNA methyltransferases (*DNMT1*, *DNMT3a* and *DNMT3b*) were hypermethylated in mesothelioma cells in comparison to normal pleura, a result that was further confirmed in the TCGA cohort (203). More recently, through genome-wide methylation array technology Guarrera et al. analyzed the methylome of 163 MPM cases whose exposure to asbestos was previously assessed in comparison to control samples (202). Here, the authors identified differential profiles of DNA methylation, 98% of which comprised hypomethylated single-CpG. These genomic regions were mainly associated with genes involved in immune systems processes. These profiles were not histotype-specific, a part from the couple *CXCR6* and *FYCO1* which had lower methylation level in biphasic mesotheliomas than epithelioid. However, the striking result was the most significant hypomethylated CpG in 7p22.2 associated with the Forkhead Box K1 (*FOXK1*), a transcription factor involved in pathways like development and metabolism, and, most of all, directly interacting with *BAP1*. It has been suggested that the dephosporylation of *FOXK1* transactivated CCL-2 gene and promotes the activation of TAMs (204). These recent evidences push to go further in the investigation of these markers, especially considering their involvement in the immune system processes that make them a likely target for immunotherapy.

The identification of DNA methylation as a marker in blood has also been explored so far. The most studied gene is mesothelin encoding the mesothelin-related peptide (SMRP) which is generally methylated in the normal pleura, while it is modified in MPM. However, it has a low sensitivity to be considered as a good marker for MPM (204). This led Santarelli et al. to identify a new marker for asbestos-exposed mesotheliomas (194). Thrombomodulin (TM) expression is silenced in malignant mesothelioma through a mechanism that involved the methylation of TM promoter by the recruitment of PARP1. Since the methylation of TM promoter has been associated with survival and given the role of PARP1 in the methylation mechanism, this marker may be of interest for further investigation for therapeutic development. In 2019, Cugliari et al. analyzed the peripheral blood of 159 MPMs and identified the CpG dinucleotide cg03546163 region associated with the gene *FKBP5* as a significant marker for prognosis (205). This is very interesting as *FKBP5* increases chemosensitivity to the AKT pathway, which is druggable in mesothelioma as previously described.

Other epigenetic modifications involve the acetylation and methylation of histones. A study by Goto et al. has identified a high expression of state of histone H3 lysine methylation (H3K27me3) mark (206). Interestingly, high expression marks of H3K27me3 have been associated with overexpression of EZH2 (207). This last has been found as a marker of poor prognosis in mesothelioma (207). Moreover, preclinical studies have shown that loss of function in BAP1 make cells sensitive to the inhibitors of EZH2. Among them, tazemetostat, a first-in-class small-molecule inhibitor of EZH2 received approval from the FDA in January 2020 for the treatment of locally advanced or metastatic sarcoma (207), while an ongoing clinical trial named NCT02860286 has shown antitumor activity of tazemetostat in a cohort of 74 patients lacking BAP1 (128). However, the use of drugs targeting the epigenome has already been attempted in mesothelioma with negative results. In fact, in the phase III VANTAGE-014 trial the efficacy of vorinostat, a histone deacetylase inhibitor, was tested against placebo with no improvement in overall survival (135) (**Table 2**). As claimed by Garassino et al., the main biases of this study were the random selection of patients irrespective of clinicopathological features and the rapid development into a phase III trial (208). These results do not undermine the use of drugs targeting the epigenome, instead they underline the necessity to improve patients’ stratification previous to enrollment into clinical trials. In summary, the investigation of potentially reversible modifications like the epigenetics markers seem to be relevant in MPM, and, when identified through liquid biopsy, they could represent a novel and promising approach for diagnosis and monitoring of cancer progression.

## Conclusion

In this review, we have covered multiple aspects of mesothelioma microenvironment that have played and will play an important role for immunotherapeutic approaches. To this, targeting the TME with anti-PD1 (nivolumab) and anti-CTLA-4 (ipilimumab) has revealed as the most effective strategy in this disease with few therapeutic options (209). In addition, genomic and transcriptomic have allowed the identification of druggable features, currently under evaluation alone or in combination with immunotherapies. Moreover, microRNAs expression has shown a role in a better understanding of the biology of MPM, and promising preliminary data suggests a possible application in the clinic for diagnosis and monitoring as epigenetic studies.

In conclusion, a comprehensive knowledge of MPM biological aspects is crucial for a deeper understanding of such complex disease and for the improvement of patients’ outcome.

## Author Contributions

SH and LM: conceptualization, original draft preparation, and writing. AC-F and FG: conceptualization, original draft preparation, writing, and supervision. MK: conceptualization and writing. All the authors: review and editing. All authors contributed to the article and approved the submitted version.

## Conflict of Interest

The authors declare that the research was conducted in the absence of any commercial or financial relationships that could be construed as a potential conflict of interest.

## Abbreviations

ADCC, antibody-dependent cellular cytotoxicity; APC, antigen presenting cells; BAP1-TPDS,BAP1-tumor predisposition syndromes; BLM, Bloom syndrome gene; CCL2, C-C chemokine ligand 2; CCL4, C–C chemokine ligand 4; CCL5, C–C chemokine ligand 5; CSF-1R, colony stimulating factor-1 receptor; CXCL12, C-X-C Motif Chemokine Ligand 12 ; DAMPs, damage-associated molecular patterns; DC, dendritic cells; DDR, DNA damage response; DSBs, Double Strand Breaks; EMT, epithelial to mesenchymal transition; EZH2, enhancer of zeste homolog 2; FISH, fluorescent in-situ hybridization; FOXK1, Forkhead Box K1; Gr-MDSC, granulocytic myeloid-derived suppressor cells; H3K27me3, histone H3 lysine methylation; HMGB1, high mobility group protein B1; HR, Homologous recombination; HRD, Homologous Recombination Deficiency; IHC, immunohistochemistry; ILC, Innate lymphoid cells; LAG-3, lymphocyte activation gene-3; M-MDSC, monocytic myeloid-derived suppressor cells; MDSC, myeloid-derived suppressor cells; MGMT, O6-methylguanine-DNA methyltransferase; MLPA, multiplex ligation–dependent probe amplification; MMR, Mismatch Repair; MPM, malignant pleural mesothelioma; Mpseq, Mate-pair sequencing; NER, Nucleotide Excision Repair; NF2, neurofibromatosis type 2; NFKB, Nuclear Factor Kappa B; NGS, next generation sequencing; NHEJ, non-homologous end-joining; NK, natural killer; NKT, Natural killer T; NO, nitric oxide; NSCLC, non-small cell lung cancer; PARP, poly(ADP-ribose) polymerase; PARPi, PARP-inhibitors; PGE2, prostaglandin E2; RAGE, receptor for Advanced Glycation Endproducts; ROS, reactive oxygen species; SCNA, somatic copy-number alteration; SLFN11, Schlafen 11; SMRP, mesothelin-related peptide; SSBs, Single Strand Breaks; TAMs, tumor-associated macrophages; TCGA, The Cancer Genome Atlas; TEAD, transcriptional enhanced associate domain; TERT, telomerase reverse transcriptase; TGF-β, Transforming Growth Factor β; TIGIT, T cell immunoglobulin and ITIM domain; TILs, tumor infiltrating lymphocytes; TIM-3, T cell immunoglobulin and mucin-domain containing-3; TLS, tertiary lymphoid structures; TM, Thrombomodulin; TME, tumor microenvironment; Treg, Regulatory T cells; VISTA, V-domain Ig suppressor of T cell activation; αGC, glycolipid α-galactosylceramide

## Glossary

ADCC: antibody-dependent cellular cytotoxicity
APC: antigen presenting cells
BAP1-TPDS: BAP1-tumor predisposition syndromes
BLM: Bloom syndrome gene
CCL2: C-C chemokine ligand 2
CCL4: C–C chemokine ligand 4
CCL5: C–C chemokine ligand 5
CSF-1R: colony stimulating factor-1 receptor
CXCL12: C-X-C Motif Chemokine Ligand 12
DAMPs: damage-associated molecular patterns
DC: dendritic cells
DDR: DNA damage response
DSBs: Double Strand Breaks
EMT: epithelial to mesenchymal transition
EZH2: enhancer of zeste homolog 2
FISH: fluorescent in-situ hybridization
FOXK1: Forkhead Box K1
Gr-MDSC: granulocytic myeloid-derived suppressor cells
H3K27me3: histone H3 lysine methylation
HMGB1: high mobility group protein B1
HR: Homologous recombination
HRD: Homologous Recombination Deficiency
IHC: immunohistochemistry
ILC: Innate lymphoid cells
LAG-3: lymphocyte activation gene-3
M-MDSC: monocytic myeloid-derived suppressor cells
MDSC: myeloid-derived suppressor cells
MGMT: O6-methylguanine-DNA methyltransferase
MLPA: multiplex ligation–dependent probe amplification
MMR: Mismatch Repair
MPM: malignant pleural mesothelioma
Mpseq: Mate-pair sequencing
NER: Nucleotide Excision Repair
NF2: neurofibromatosis type 2
NFKB: Nuclear Factor Kappa B
NGS: next generation sequencing
NHEJ: non-homologous end-joining
NK: natural killer
NKT: Natural killer T
NO: nitric oxide
NSCLC: non-small cell lung cancer
PARP: poly(ADP-ribose) polymerase
PARPi: PARP-inhibitors
PGE2: prostaglandin E2
RAGE: receptor for Advanced Glycation Endproducts
ROS: reactive oxygen species
SCNA: somatic copy-number alteration
SLFN11: Schlafen 11
SMRP: mesothelin-related peptide
SSBs: Single Strand Breaks
TAMs: tumor-associated macrophages
TCGA: The Cancer Genome Atlas
TEAD: transcriptional enhanced associate domain
TERT: telomerase reverse transcriptase
TGF-β: Transforming Growth Factor β
TIGIT: T cell immunoglobulin and ITIM domain
TILs: tumor infiltrating lymphocytes
TIM-3: T cell immunoglobulin and mucin-domain containing-3
TLS: tertiary lymphoid structures
TM: Thrombomodulin
TME: tumor microenvironment
Treg: Regulatory T cells
VISTA: V-domain Ig suppressor of T cell activation
αGC: glycolipid α-galactosylceramide
